# An unexpected cause of dysuria in a cat in Poland

**DOI:** 10.1186/s12917-022-03493-0

**Published:** 2022-11-12

**Authors:** Marta Miszczak, Oliwia Wyleżoł, Paulina Prorok, Karolina Bierowiec

**Affiliations:** 1 Faculty of Veterinary Medicine, Department of Epizootiology and Clinic of Birds and Exotic Animals, Division of Infectious Diseases and Veterinary Administration, Wrocław University of Environmental and Life Sciences, Wrocław, Poland; 2 Faculty of Veterinary Medicine, Department of Epizootiology and Clinic of Birds and Exotic Animals, Students’ Scientific Society EZA, Wrocław University of Environmental and Life Sciences, Wrocław, Poland

**Keywords:** *Capillaria*, Cat, Bladder capillariosis, Treatment, Behaviour, House soiling

## Abstract

**Background:**

Urinary tract infestation by *Capillaria* spp. in domestic cats is rather rare, but can cause clinical symptoms and affect behaviour. To our knowledge, this report is the first to describe a case of urinary capillariosis in a cat in Poland.

**Case presentation:**

A female formerly stray cat aged about 1.5 years showing dysuria, stranguria, periuria and lethargy was presented at the veterinary clinic. Urinalysis revealed the presence of *Capillaria plica* eggs in the sediment. The cat was treated successfully with three topical doses of Broadline (Merial, Toulouse, France).

**Conclusions:**

*C. plica* is a nematode whose definitive hosts are carnivores, which are infected by eating earthworms (the intermediate hosts). Thus, *C. plica* infestation is more frequent in wild carnivores and dogs, and rare in cats. Symptomatic bladder capillariosis in cats is very rarely diagnosed and described.

## Background

Urinary bladder infections caused by *Capillaria plica* occur worldwide in wild canids (e.g. red foxes, wolves, badgers) and wild cats (*Felis silvestris*) [1, 2], but symptomatic capillariosis in domestic animals is rarely reported [3]. Only a few cases of bladder infection caused by *Capillaria* spp. in domestic cats have been described [1, 4–6], although post-mortem surveys conducted in Germany and Australia have revealed that the incidence of infestation with this parasite in cats ranges from 6 to 18.3% [3, 7]. Most described cases of symptomatic infestation in domestic cats are with *C. (Pearsonema) plica*, but *Capillaria feliscati* and *Capillaria travassoi* infestations also occur [8]. Whether these species should be considered distinct or the same species in different hosts remains unclear [7, 9].

*C. plica* is a small (13–60 mm long and 0.048–0.09 mm wide), yellowish, threadlike nematode [4]. Carnivores are its definitive hosts and earthworms are its intermediate hosts [3]. The final host is infected orally by ingesting the worm’s larvae (stage L1). Then, the larvae spend 2 months in the intestine (stages L2 and L3) [2, 3] and move to the bladder, where they reach sexual maturity. Thereafter, the parasite penetrates the bladder mucosa, urethra or renal pelvis [2, 3]. The grey, barrel-shaped eggs laid by female worms (55–67 × 26–29 μm) are spread into the environment with urine. The pre-patent period is 58–63 days. Infection is not typically associated with any clinical symptom and is diagnosed incidentally during urine examination [1]. Rarely, infected animals have been reported to show signs of lower urinary tract disease, such as haematuria, pollakiuria, stranguria and dysuria [1–3]. Other reported clinical signs include abdominal pain, fever, urinary incontinence, straining and cystitis requiring appropriate treatment. Only causative treatment with anthelmintics brings lasting improvement of clinical signs [8]. In cats, urinary capillariosis is usually detected incidentally via the observation of the parasite’s eggs in urine samples. We believe that a reminder that this parasite can cause painful symptoms in cats is very important for veterinary clinicians.

## Case presentation

A crossbreed spayed female cat aged about 1.5 years was submitted to the veterinary clinic in September 2020. During the diagnostic interview, the owners reported that the cat was a recently adopted stray. From its first day in its new home, the cat had problems with urination, which was the reason for the owners’ consultation with a veterinary behaviourist. It released urine outside of the litterbox onto horizontal surfaces such as beds, pillows, blankets and carpets, and the owner had observed that urination seemed to be painful and the cat was straining to urinate. The cat was also lethargic and reluctant to play. The cat was first referred to a veterinary clinician for examination to exclude somatic causes of urination outside the litter box.

During the veterinary consultation, the owner reported only the cat’s problem with urination outside of the litterbox. The veterinary clinician conducted an anamnesis and full clinical examination of the cat, including full physical examination, urinalysis (including sediment assessment and bacteriological culture), morphological and biochemical blood testing, ultrasound examination of the kidneys and urinary system and a rapid one-step test for feline infectious diseases [i.e. feline immunodeficiency virus (FIV) and feline leukaemia virus (FeLV); VetExpert Rapid Test FIV Ab/ FeLV Ag; VetExpert, Łomianki, Poland]. Clinical examination revealed a body condition score of 4/9, pink and moist mucous membranes, painless and non-enlarged peripheral lymph nodes, a soft and painless abdomen, a physiological murmur upon auscultation of the trachea and pulmonary fields, a regular heart rhythm with no murmur or additional tone and a normal body temperature (38.1 °C). The rapid test for antibodies against FIV and FeLV antigens yielded negative results. The haematology and serum chemistry findings are presented in Table 1.

**Table 1:** Haematology and serum chemistry findings

	Result	Units	Reference values
**Haematology**			
WBC	8.2	10^3^/μl	5.5 – 19.0
Lym	3.4	10^3^/μl	1.0 – 5.0
Mono	0.7	10^3^/μl	0.1 – 1.0
PMN	4.1	10^3^/μl	2.0 – 8.0
Lym%	41.4	%	20.0 – 55.0
Mono%	8.0	%	1.0 – 4.0
PMN%	50.6	%	35.0 – 75.0
RBC	8.16	10^6^/μl	6.50 – 10.00
HGB	12.3	g/dl	8.0 – 15.0
HCT	32.5	%	24.0 – 45.0
MCV	39.8	μm^3^	39.0 – 55.0
MCH	15.1	pg	13.0 – 17.0
MCHC	37.8	g/dl	30.0 – 36.0
RDWC	20.2	%	10.0 – 16.0
RDWS	29.2	μm^3^	37.0 – 46.0
PLT	854	10^3^/μl	100 – 400
MPV	9.6	μm^3^	7.0 – 11.0
**Serum chemistry**			
Albumine (Alb)	35.7	g/l	26.0 – 46.0
Alanine transaminase (ALT)	53.2	U/l	1.0 – 91.0
Alkaline phosphatase (Alk Phos)	57.0	U/l	1.00 – 140.0
Aspartate transaminase (AST)	21.3	U/l	1.00 – 59.0
Total protein (TP)	70.5	g/l	57.0 – 94.0
Glucose (Glu)	6.13	mmol/l	3.05 – 6.10
Creatinine (Cr)	97.4	μmol/l	1.00 – 168.0
Blood urea nitrogen (BUN)	6.03	mmol/l	5.00 – 11.3
Albumin/globulin ratio (A/G)	1.03		0.500 – 1.10
Globulin (G)	34.8	g/l	19.0 – 66.0

**Fig. 1:**
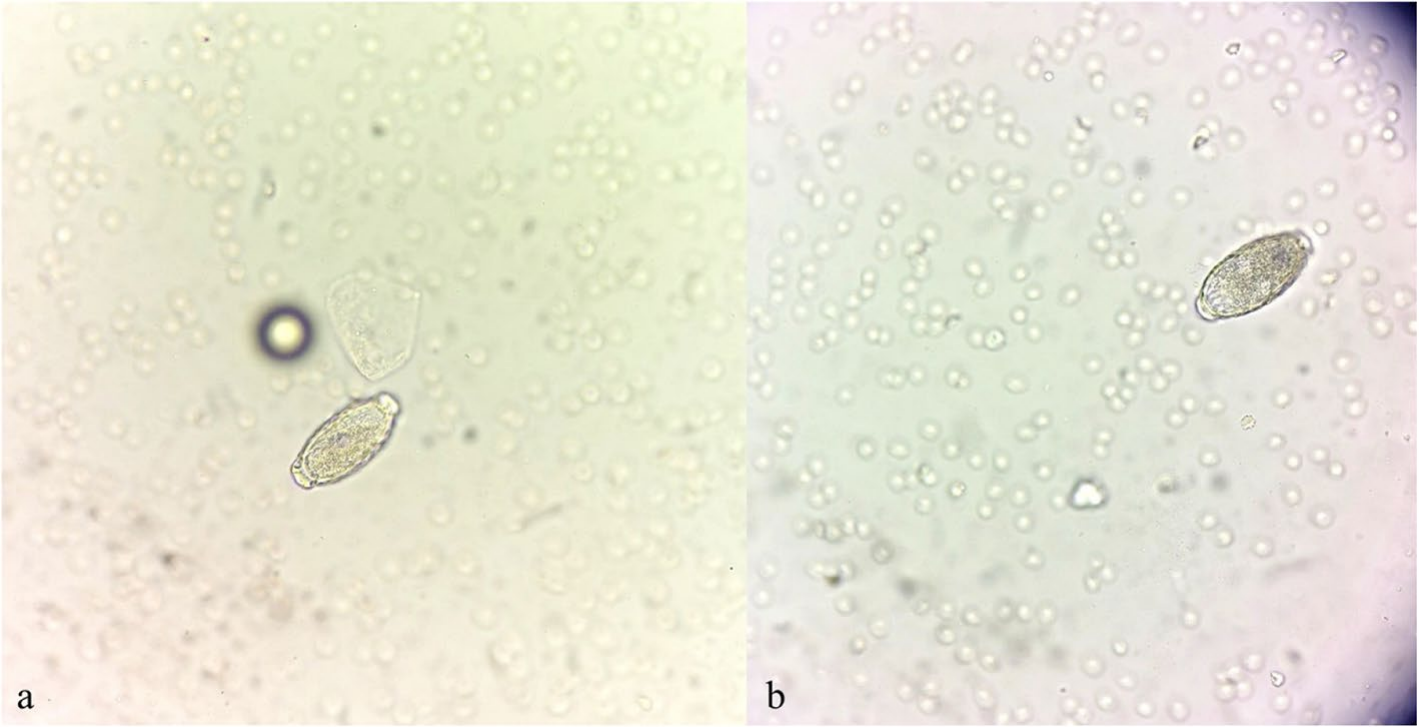
**a, b** ‘Barrel-shaped’ *Capillaria plica* eggs in urine sediment from the described cat. The eggs have slightly pitted shells and two opercules with polar plugs (40× magnification; photos taken by Karolina Jakubiak, DVM)

The ultrasound examination revealed no abnormality; the bladder was filled with moderately echogenic urine with no visible foreign body or crystals, the bladder wall was smooth and 0.3 cm thick, the urethra was not dilated, the kidneys were of normal size with a preserved cortex-core structure and the renal pelvis was not dilated. Laboratory tests revealed a urine specific gravity of 1.015 g/L (pH 7.5) and proteinuria. A urine sample was taken by cystocentesis, followed by immediate centrifugation and assessment of the urine sediment in the veterinary clinic. *C. plica* eggs accompanied by erythrocytes and leukocytes were observed in the sediment (Fig. 1). Another urine sample was sent for bacteriological culture in an external veterinary laboratory (VETLAB, Wrocław, Poland). As the culture revealed no bacterial growth, no antibiogram was performed. In addition, the veterinarian performed faecal flotation, and no parasite or egg was detected in the material. On the basis of the clinical symptoms and urine analysis results, inflammation of the urinary bladder resulting from *C. plica* infection was diagnosed. However, given that the cat had been adopted from the street, with a significant change in its living environment and disruption of its routine, a more complex cause of its symptoms including behavioural disorders could not be ruled out.

On the basis of the latest scientific reports, a spot-on topical combination formulation of fipronil (8.3% wt/vol), S-methoprene (10% wt/vol), eprinomectin (0.4% wt/vol) and praziquantel (8.3% wt/vol; Broadline; Merial, Toulouse, France) was administered three times at 14-day intervals [8]. Each round of treatment was preceded by urinalysis. Samples were obtained by cystocentesis and examined with a URIT-50 Urine Analyser (Urit Medical Electronic, Guangxi, China); the results are presented in Table 2. Additionally, some of the behaviourist’s recommendations were implemented to improve the cat’s well-being and reduce unwanted behaviour. To prevent urination outside of the litterbox and ensure the provision of comfortable conditions for the cat, the owners provided a larger litterbox and additional litterboxes in locations of previous accidental urination. They also diversified the cat’s environment by adding observation sites (wall shelves) at high locations, scratching surfaces (sisal scratching posts and boards) to provide the cat with scratching opportunities and to encourage it to leave pheromones from the plantar pad glands in the apartment (increasing comfort), and attractive toys to encourage hunting behaviour and improve the cat’s mood. In addition, the veterinary clinician prescribed dietary supplements aimed at improving the cat’s mood and mental well-being [a calming syrup containing L-tryptophan, L-theanine and vitamins (Relaxer VET PLUS; Scanvet Poland, Gniezno, Poland) and capsules supporting urinary tract function and reducing the urine pH (UrinoVet Cat Dilution; VetExpert, Łomianki, Poland)].

**Table 2:** Results of urinalyses performed during treatment

	No 1	No 2	No 3	No 4
	Day 0 (before treatment)	Day 14 (after the first dose of Broadline)	Day 28 (after the second dose of Broadline)	Day 42 (after the third dose of Broadline)
Leucocytes	+++ (70 cells/μL)	+++ (500 cells/μL)	+++ (500 cells/μL)	+++ (500 cell/μL)
Protein	+ (0.3 g/L)	+ (0.3 g/L)	+ (0.3 g/L)	++ (1.0 g/L)
pH	7.5	7.0	7.0	6.5
Specific Gravity (g/l)	1.015	1.020	1.020	1.030
Urobilinogen (μmol/L)	33	normal	normal	normal
Vc (mmol/L)	1.4	0.6	0.5	5.6
Ketones	–	–	–	–
Nitrite	–	–	–	–
Bilirubine	–	–	–	–
Glucose	–	–	–	–
Blood	–	+/− (10 cells/μL)	+/− (10 cells/μL)	–
Sediment	*Capillaria plica* eggs	no eggs found	no eggs found	no eggs found
	leukocytes (0-2/hpf)	leukocytes (0-2/hpf)	leukocytes (0-2/hpf)	leukocytes (0-2/ hpf)
	erythrocytes (0-3/hpf)	erythrocytes (0-3/hpf)	erythrocytes (0-3/hpf)	
	single epithelial cells			
	numerous lipid droplets		single struvites	
Urine culture	negative	–	negative	–

After the first administration of the drugs and the introduction of the environmental modifications, the cat ceased urinating outside of the litterbox, but the owner observed that it still had a strained posture during urination. Urinalyses performed after the initiation of Broadline treatment revealed no *C. plica* egg, but the third test revealed struvite crystals. Thus, an additional bacteriological urine test was ordered, and the results were negative. The veterinarian replaced the cat’s previously administered dry food with a moist urological food (Kattovit Feline Diet Urinary; FINNERN GmbH & Co. KG, Verden, Germany) to improve hydration and help to reduce the urine pH, thereby preventing the development of bacterial bladder infection associated with the presence of crystals and an alkaline pH [3]. As the cat still showed discomfort or pain during urination, oral amitriptyline hydrochloride (Amitriptylinum VP; Bausch Health Poland; ICN Polfa Rzeszów, Rzeszów, Poland) (5 mg once a day for 1 month) was administered due to its expected analgesic, anti-inflammatory and anticholinergic effects [10]. The cat’s problematic behaviour and straining in the litterbox resolved, and the owner reported that they had not recurred during a follow-up telephone consultation 4 weeks later.

## Discussion and conclusions

Data on *C. plica* infection in domestic cats in Europe and worldwide are very limited [11]. The parasite is widespread, especially in the red fox population [2, 3], which has begun to inhabit suburbs and city centres. This situation favours more frequent parasitemia after earthworm consumption in by city dogs, hunting dogs and stray or outdoor cats. Urinary capillariosis is rarely reported in dogs and cats [12–16]. In both species, it can be self-limiting with no clinical sign [2, 3]; thus, the actual incidence of bladder capillariosis can be assumed to be underestimated. Severe infection can cause abdominal pain, fever, lethargy, reduced appetite, dysuria, periuria, pollakiuria and haematuria [3, 5, 6, 17]. Feline capillariosis may be associated with painful obstruction caused by adult nematodes in the ureter or renal pelvis, or urethral inflammation with submucosal oedema, and may lead to acute renal failure or predisposition to the development of feline urinary tract disease [5]. Wilson-Hanson and Prescott [7] reported the presence of inflammatory changes (areas of dilated blood vessels, extravasated blood and inflammatory cells) in the bladders of cats with urinary capillariosis, although none of the observed mature *C. plica* appeared to be attached to the mucosa (they floated freely in the urine). In another study, mature *C. plica* were observed to be attached superficially or buried within the mucosa [6]. In dogs, *C. plica* infection may lead to renal failure with chronic interstitial nephritis, glomerular amyloidosis, and chronic inflammation of the bladder and renal pelvis with the infiltration of mononuclear cells into the submucosa [17].

In the present case, ultrasound examination of the cat’s urinary system revealed no mature parasite attached to the bladder mucosa or urethral obstruction, and no histopathological examination of the bladder wall samples was performed. Thus, whether the presence of parasites in the urinary tract was the main cause of the clinical symptoms and, if so, what inflammatory changes they caused could not be determined. However, reports suggest that the role of these parasites in inducing inflammation and clinical signs in cats should be taken into account, even when mature parasites are not seen on ultrasound [6, 7]. In the present case, crystalluria could have been an additional factor explaining the clinical signs; identification of the factor having the greatest impact on the onset of clinical symptoms was difficult. Apart from the obvious importance of medical conditions, numerous risk factors have been proposed for general urinary house soiling and latrine-related behaviour; they include anxiety, significant social or environmental changes, the owner’s absence and poor quality of the litter and litterbox [18]. The International Society of Feline Medicine emphasises that four basic factors should be considered when diagnosing the causes of feline house soiling: medical aetiologies, feline idiopathic cystitis (FIC), aberrant marking behaviour and elimination related to primary environmental or social factors [19]. As these causative factors may coexist, an optimum diagnostic approach including urinalysis (with urine sediment examination), urine culture, abdominal ultrasound and radiography is recommended [19]. When all diagnostic parameters are within normal limits, medical causes of house soiling are unlikely, but the patient could still have FIC [19]. In the present case, the significant change in the cat’s living environment and severe stress alone could be responsible for the occurrence of undesirable behaviour. The veterinary clinician treated the parasitic infection and symptoms of cystitis as well as the cat’s disturbed mental well-being resulting from adaptation difficulties**.** As a result, the determination of which of the administered drugs and supplements and applied environmental modifications had a decisive influence on the course of therapy and its final satisfactory effect was not possible. However, *C. plica* eggs were not observed in urine samples obtained after the administration of the first antiparasitic dose, reflecting the effectiveness of this treatment, as described previously by Knaus et al. [8].

The successful treatment of *C. plica* infection with fenbendazole, benzimidazoles, ivermectin, levamisole, moxidectin-imidacloprid and milbemycin has been reported [2, 3, 5, 6, 12, 14]. The Broadline (Merial) topical treatment is highly effective and is delivered in spot-on form, which is easier than oral administration and increases the chance that the owner will successfully apply the correct dose. Cats that previously lived on the street tend to be less (or not at all) socialised toward humans, which may make manipulation in the oral cavity extremely difficult or impossible to perform. This case report confirms that feline urinary capillariosis can be treated successfully with Broadline (Merial), as described previously [8].

To our knowledge, this report is the first to describe a case of *C. plica* infection in a domestic cat in Poland. Although *C. plica* infection in cats is rarely reported, clinicians should be aware that it causes symptoms such as haematuria, dysuria and pollakiuria, which may indicate capillariosis. It is also important to remember that sometimes only very bothersome behaviours, such as house soiling (especially on beds, pillows and carpets), may prompt owners to visit a veterinarian or behaviourist. Not every owner is able to recognise the symptoms of dysuria in a cat, but the cat’s nuisance behaviour quickly prompts them to seek help from a specialist.

## Data Availability

All data generated in this study are provided in this article.

## Abbreviations

FeLV: Feline leukaemia virus
FIC: Feline idiopathic cystitis
FIV: Feline immunodeficiency virus
hpf: High power field
wt/vol: Weight/volume
Vc: Ascorbic acid
